# Full Genome of batCoV/MinFul/2018/SriLanka, a Novel Alpha-Coronavirus Detected in *Miniopterus fuliginosus*, Sri Lanka

**DOI:** 10.3390/v14020337

**Published:** 2022-02-07

**Authors:** Therese Muzeniek, Thejanee Perera, Sahan Siriwardana, Dilara Bas, Fatimanur Kaplan, Mizgin Öruc, Beate Becker-Ziaja, Inoka Perera, Jagathpriya Weerasena, Shiroma Handunnetti, Franziska Schwarz, Gayani Premawansa, Sunil Premawansa, Wipula Yapa, Andreas Nitsche, Claudia Kohl

**Affiliations:** 1 Centre for Biological Threats and Special Pathogens, Highly Pathogenic Viruses (ZBS 1), Robert Koch Institute, 13353 Berlin, Germany; muzeniekT@rki.de (T.M.); basD@rki.de (D.B.); kaplanF@rki.de (F.K.); oerucM@rki.de (M.Ö.); schwarzF@rki.de (F.S.); nitscheA@rki.de (A.N.); 2 Institute of Biochemistry, Molecular Biology and Biotechnology, University of Colombo, Colombo 00300, Sri Lanka; thejanee90@gmail.com (T.P.); jagath@ibmbb.cmb.ac.lk (J.W.); shiromah@ibmbb.cmb.ac.lk (S.H.); 3 IDEA (Identification of Emerging Agents) Laboratory, Department of Zoology and Environment Sciences, University of Colombo, Colombo 00300, Sri Lanka; sahan@zoology.cmb.ac.lk (S.S.); icperera@sci.cmb.ac.lk (I.P.); suviprema@gmail.com (S.P.); wipula@gmail.com (W.Y.); 4 Centre for International Health Protection, Public Health Laboratory Support (ZIG 4), Robert Koch Institute, 13353 Berlin, Germany; becker-ZiajaB@rki.de; 5 Colombo North Teaching Hospital, Ragama 11010, Sri Lanka; gavisprema@gmail.com

**Keywords:** bat alphacoronavirus, *Miniopterus fuliginosus*, Sri Lanka, cave-dwelling, sympatric colony, full genome, coronavirus, one health

## Abstract

Coronaviruses (CoV) are divided into the genera α-CoVs, β-CoVs, γ-CoVs and δ-CoVs. Of these, α-CoVs and β-CoVs are solely capable of causing infections in humans, resulting in mild to severe respiratory symptoms. Bats have been identified as natural reservoir hosts for CoVs belonging to these two genera. Consequently, research on bat populations, CoV prevalence in bats and genetic characterization of bat CoVs is of special interest to investigate the potential transmission risks. We present the genome sequence of a novel α-CoV strain detected in rectal swab samples of *Miniopterus fuliginosus* bats from a colony in the Wavul Galge cave (Koslanda, Sri Lanka). The novel strain is highly similar to Miniopterus bat coronavirus 1, an α-CoV located in the subgenus of Minunacoviruses. Phylogenetic reconstruction revealed a high identity of the novel strain to other α-CoVs derived from Miniopterus bats, while human-pathogenic α-CoV strains like HCoV-229E and HCoV-NL63 were more distantly related. Comparison with selected bat-related and human-pathogenic strains of the β-CoV genus showed low identities of ~40%. Analyses of the different genes on nucleotide and amino acid level revealed that the non-structural ORF1a/1b are more conserved among α-CoVs and β-CoVs, while there are higher variations in the structural proteins known to be important for host specificity. The novel strain was named batCoV/MinFul/2018/SriLanka and had a prevalence of 50% (66/130) in rectal swab samples and 58% (61/104) in feces samples that were collected from Miniopterus bats in Wavul Galge cave. Based on the differences between strain batCoV/MinFul/2018/SriLanka and human-pathogenic α-CoVs and β-CoVs, we conclude that there is a rather low transmission risk to humans. Further studies in the Wavul Galge cave and at other locations in Sri Lanka will give more detailed information about the prevalence of this virus.

## 1. Introduction

Coronaviruses (CoVs) are the members of the family Coronavirinae within the order Nidovirales, and they can cause respiratory diseases in animals and humans [1]. CoVs have positive-sense, single-stranded RNA genomes with sizes between 27,000 and 32,000 nucleotides (nt) [2]. In general, the genome is organized in five major open reading frames (ORF), encoding a number of non-structural proteins for viral replication (ORF1a/1b), and the genes for the structural proteins of spike (S), membrane (M), envelope (E) and nucleocapsid (N) protein [3]. While the ORF1a/1b-coding sequences (CDS) are generally considered as highly conserved gene sections since they need to maintain protein functionality, the structural proteins are rather susceptible to substitutions on gene and protein levels. These variations, especially in the spike protein, can lead to differences in their infectivity and host specificity. In general, CoVs can be divided into the genera of α-CoVs, β-CoVs, γ-CoVs and δ-CoVs [4]. While γ-CoVs and δ-CoVs are probably derived from bird CoVs, α-CoVs and β-CoVs can be found in a variety of bat species, which are being discussed as their potential natural reservoir [5]. These two genera are also prevalent in a wide range of other mammals (wildlife and domestic animals) and humans. Viruses of the genus β-CoV such as SARS-CoV, MERS-CoV and SARS-CoV-2 can cause severe respiratory symptoms in humans, and their easy spread among humans holds the proven risk of pandemic developments. With the latest emergence of SARS-CoV-2 there are seven known CoVs that are capable of infecting humans [6]. α-CoVs are also capable of causing infections in humans; HCoV-229E and HCoV-NL63 cause mild upper respiratory diseases in humans and appear seasonally [7]. A thorough genomic characterization and understanding of the genotypical and phenotypical differences between α-CoVs and β-CoVs can help to provide understanding of the human-pathogenic potential of different CoVs.

In Sri Lanka, a high biodiversity in general and in particular a high bat species variety can be observed [8]. Although some of the 30 different bat species in Sri Lanka are described to be a potential reservoir for CoVs in other countries, there is only little knowledge on the prevalence of chiroptera-hosted CoVs on the Sri Lankan island so far [9]. In this study, a population of different bat species roosting in one of the largest natural caves (Wavul Galge, Koslanda, Sri Lanka) was examined. In a previous study, we have reported the detection of novel α-CoV fragments in feces and rectal swabs from a number of *M. fuliginosus* bats [10]. In this study, we present the first full genome of an α-CoV detected in Sri Lankan bats.

## 2. Materials and Methods

This study was carried out according to the relevant guidelines and regulations of the Fauna and Flora Protection Ordinance, approved by the local government authority (Department of Wildlife Conservation, Sri Lanka, permit No. WL/3/2/05/18, issued on 10 January 2018).

### 2.1. Bat Sampling

Sampling of cave-dwelling bats roosting in the Wavul Galge cave (Sri Lanka) was performed in March and July 2018 and January 2019 as described before [10]. Adequate personal protective equipment such as gloves, safety glasses and FFP3 masks were worn during the capturing and sampling procedure. All lab procedures were conducted under Biosafety level-2 conditions with appropriate precautions.

Bats were captured by using hand nets when leaving the cave at nightfall and were kept in bat holding bags until further processing. Bat species were determined by using macroscopic identifiers and documented together with other features such as weight, forearm length, sex and stage of age. For molecular species identification based on the cytochrome B gene, an oral swab was taken from each bat. Furthermore, fresh fecal pellets were collected with forceps from the holding bags if available, or else a rectal swab was taken. Samples were snap-frozen by using liquid nitrogen before storage at −80 °C until further processing.

In all three sampling sessions (March and July 2018, January 2019) a total of 395 bats were sampled, all belonging to the genera Miniopterus, Rousettus, Hipposideros and Rhinolophus (based on macroscopic species identification).

### 2.2. Shotgun NGS

For processing of rectal swabs, 500 µL of sterile PBS were added and mixed by vortexing. After a centrifugation step, 140 µL of the supernatant was used for extraction with a viral RNA mini kit (QIAGEN, Hilden, Germany).

To obtain the full genome of a bat α-CoV, 65 rectal swabs from sampling session July 2018 (only *M. fuliginosus*) were prepared for shotgun NGS. Before further processing, 5 to 10 RNA samples were pooled. Pools were digested at 37 °C for 30 min by using the TURBO DNA-free Kit (Invitrogen, Carlsbad, CA, USA) following the manufacturer’s protocol. For cDNA synthesis, SuperScript IV Reverse Transcriptase (Invitrogen, Carlsbad, CA, USA) and random hexamer primers were used. The cDNA was used for second strand synthesis by using the NEBNext^®^ Ultra™ II Non-Directional RNA Second Strand Synthesis Module (New England Biolabs, Ipswich, MA, USA) following the manufacturer’s instructions. dsDNA was purified by using the Agencourt AMPure XP bead system (Beckman Coulter Life Sciences, Krefeld, Germany), adding 120 µL of magnetic beads per sample for binding, followed by two washing steps with 200 µL of 70% ethanol and an elution in 40 µL of PCR grade water. DNA concentration was determined by using a NanoDrop™ 1000 Spectrophotometer (Thermo Fisher Scientific, Hennigsdorf, Germany). Samples were sequenced on a HiSeq 2500 sequencer (Illumina, San Diego, CA, USA) with a paired end read output of 2 × 250 bp and a total output of up to 7.5 million reads per pool.

### 2.3. NGS Data Analysis and Full Genome Assembly

Trimmed data were analyzed by using a diamond tool [11] and BLASTx algorithm with the “- - sensitive” setting. BLAST results were visualized in MEGAN [12] and Geneious Prime software (version 2020.2.3, Biomatters Ltd., Auckland, New Zealand).

In order to obtain the full genome sequence of the novel bat coronavirus, reads of all rectal swab pools were mapped to a reference sequence (BtMf-AlphaCoV/AH2011—Accession No. KJ473795) by using Geneious Prime software. The consensus sequence was calculated from the final assembly by using Geneious Prime software with distinct quality settings.

The quality of the generated consensus sequence was further validated. For areas with lower coverage or gaps in the consensus sequence, spanning primer pairs were designed based on the sequence data already available. RNA of the single rectal swab samples before pooling was transcribed to cDNA as described before and amplified with the respective spanning primers by using a standard PCR protocol (available upon request) and the Taq DNA Polymerase Kit (Invitrogen, Carlsbad, CA, USA). PCR products were analyzed by using agarose gel electrophoresis. Positive PCR products with distinct bands visible in the agarose gel were Sanger sequenced by using the BigDye Terminator Cycle Sequencing Kit on an Applied Biosystems 3500 Dx Genetic Analyzer (Applied Biosystems, Waltham, MA, USA) with the respective forward and reverse primers.

PCR products with multiple bands were purified by using the MSB Spin PCRapace Kit (Invitrogen, Carlsbad, CA, USA) and sequenced by using MinION sequencing (Oxford Nanopore Technologies, Oxford, UK). The sequences were analyzed by using Geneious Prime software and mapped to the already existing consensus sequence as described before. After obtaining a full genome sequence, genes were annotated by using the annotation tool in the Geneious Prime software and Glimmer annotation tool [13,14,15].

### 2.4. Sequence Analysis and Phylogenetic Reconstruction

For phylogenetic reconstruction, the full genome as well as ORF1b CDS were used, respectively. A number of reference sequences were selected as representatives for the different subgenera of α-CoVs and β-CoVs and were downloaded from the NCBI database (Table 1). Recombination analysis of the full genome sequence and two closely related strains was performed using DualBrother detection software [16,17]. For phylogenetic reconstruction, nucleotide alignment of all selected representative CoV strains was calculated by using the MAFFT algorithm [18]. Phylogenetic trees were calculated by using MrBayes version 3.2.6 [19]. The model GTR with gamma-distributed rate variation was selected for the calculations, and the parameters were set as follows: number of runs: four; number of generations: 500,000 to 1,000,000; subsampling frequency: 100 and burn-in: 10%. The phylogenetic trees were visualized using Geneious prime software.

### 2.5. Real-Time RT-PCR Design

**Figure 1:**
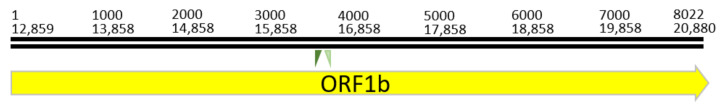
Location of the newly designed batCoV real-time RT-PCR assay (green labels) on the ORF1b gene (yellow), producing an amplicon of 121 bp at nucleotide position 16,286–16,406.

To determine the prevalence of the novel α-CoV in the examined bat population, a specific real-time RT-PCR was designed based on the obtained sequencing data. A region on the conserved ORF1b CDS (nt position 16,286–16,406) with high read coverage was selected, and a forward primer (TggTTTTTgTgTTAACATCACAT), a reverse primer (gCAAAATCACTACTAATATTgAACAC) and a probe (FAM-ACCACCTTTgAgAgCTCCTACAATCgC-BHQ1) were designed, producing a 121 bp amplicon (Figure 1). The specificity of the assay was tested in silico by mapping the primers to the reference strains (Table 1), allowing up to 5 mismatches. In addition, the specificity of the PCR assay was tested in vitro with other available samples of α-CoVs and β-CoVs. For quantification of the samples, in vitro RNA (ivRNA) of the 121 bp amplicon was synthesized (GenExpress, Berlin, Germany). Using the ivRNA, the sensitivity of the assay was validated. The detection limit was 10 copies per reaction. The final PCR protocol involved the AgPath ID Kit (Invitrogen, Carlsbad, CA, USA) with 3 µL of RNA sample. The thermal profile included a reverse transcription step at 45 °C for 15 min, followed by 90 °C for 10 min. PCR cycling was performed at 95 °C for 15 s and 60 °C for 30 s for overall 45 cycles. The ivRNA was measured in different concentrations parallel to the samples to calculate the viral load per sample.

RNAs of all rectal swab and feces pellets, collected at the three sampling points, were tested with the newly designed bat α-CoV real-time RT-PCR assay to determine the prevalence of the new strain in the samples set.

## 3. Results

In this study, nine pools containing a total of 65 rectal swab samples were sequenced and subsequently analyzed. All these samples were collected from *M. fuliginosus* bats during the sampling session in July 2018.

### 3.1. NGS, Full Genome Assembly and Gene Organization

From NGS data, a total of 7332 reads were mapped to the BtMf-AlphaCoV/AH2011 reference genome (KJ473795) that covered almost the whole genome. Fifteen gaps or low-quality areas were additionally sequenced by using the Sanger and MinION sequencing method as described before. Finally, the complete genome of the novel bat coronavirus with a length of 27,987 nucleotides was obtained. The novel strain was named batCoV/MinFul/2018/SriLanka according to the literature. The full genome sequence of the batCoV/MinFul/2018/SriLanka is available at GenBank (OL956935).

The analysis of the full genome revealed a similar gene organization as the reference genome BtMf-AlphaCoV/AH2011 and other α-CoVs from Miniopterus bats, starting with the CDS of the non-structural polyproteins ORF1a/1b, followed by the genes for the structural spike protein, envelope, membrane and nucleocapsid protein (Table 2). All CoVs are known to have a universal frame shift in the ORF1a/1b polyprotein with the sequence pattern U_UUA_AAC [20,21]. With this, a ribosomal frameshift is induced and allows the translation of the ORF1b polyprotein. The polyprotein ORF1a/1b of the novel strain batCoV/MinFul/2018/SriLanka was annotated accordingly.

In order to classify the genome of batCoV/MinFul/2018/SriLanka, comparative analysis of the complete genome and genes was performed with selected reference strains of α-CoVs and β-CoVs. Table 3 gives an overview on the pairwise identity of the novel batCoV/MinFul/2018/SriLanka with selected references on nucleotide (nt) and amino acid (aa) level.

BatCoV/MinFul/2018/SriLanka shares the highest identities with other α-CoVs from Miniopterus bats; identities among all α-CoVs used in this analysis ranged from 57.0 to 85.1% on the nucleotide level of the full genome comparative analysis. In contrast, the novel batCoV/MinFul/2018/SriLanka shared identities of 38–42.8% with the selected β-CoVs.

#### 3.1.1. Non-Structural Protein CDS: ORF1a/1b

Based on the alignments of the non-structural polyprotein CDS ORF1a/1b (Table 3), the novel batCoV/MinFul/2018/SriLanka shared the highest identities with the Miniopterus bat coronavirus 1 and the strain BtMf-AlphaCoV/AH2011. For ORF1a, calculated nucleotide identities were ≥83% (aa level: ≥86.1%). In contrast, the human-related α-CoVs 229E and NL63 showed lower nucleotide identities of around 55% (aa: 47.7%). All β-CoVs showed lower nucleotide identities of 32.4–39.9% (aa: 19.7–20.5%) compared to the α-CoVs. In general, the ORF1b segment of the non-structural ORF1a/1b polyprotein showed higher identities compared to ORF1a. Especially the comparative analysis to the β-CoVs showed identities of 57.1–59.6% on nucleotide level (aa: 53.2–56.5%), which is remarkably higher than the identities calculated for the complete genomes and for the other structural proteins.

#### 3.1.2. Structural Protein CDS: Spike, Envelope, Membrane, Nucleotide

The comparative analysis of the structural protein CDS generally reflected the results of the analysis of the full genomes. Despite this, the spike protein of all β-CoVs showed ~5% lower identities compared to the pairwise identities of the full genome. The same applies to the β-CoV sequences of the envelope and nucleocapsid proteins, while the membrane proteins showed higher identities comparable to the pairwise identities of the full genomes.

**Figure 2:**
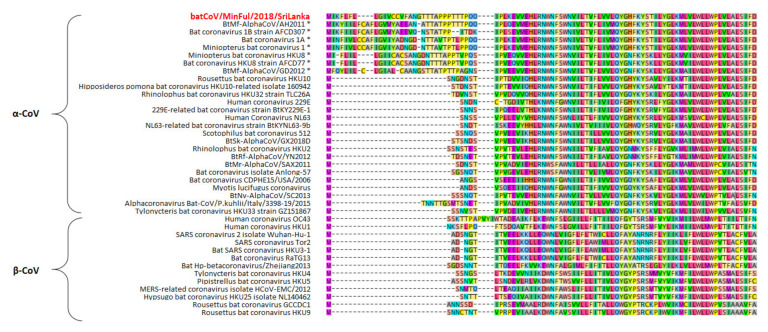
Extract of the first 100 amino acids (aa) of a multiple sequence alignment of the membrane protein from different α-CoVs and β-CoVs, calculated with MAFFT algorithm and visualized in Geneious Prime software with a color code for each aa. The novel strain batCoV/MinFul/2018/SriLanka is marked in red. α-CoVs of the subgenus Minunacovirus are marked with an asterisk.

Interestingly, the membrane proteins of the novel batCoV/MinFul/2018/SriLanka and other α-CoVs from the subgenus Minunacovirus have lengths of 253–256 aa, while the remaining α-CoVs and β-CoVs have lengths of 219–236 aa. These additional amino acids in the Minunacovirus sequences are located at the beginning of the membrane protein sequence (Figure 2).

For the spike protein sequences, the multiple alignments showed high variation in the first section (aa 1–596 of the batCoV/MinFul/2018/SriLanka spike CDS) with identities of 10% and less between α-CoV and β-CoV strains. The second section (aa 597–1375 of the batCoV/MinFul/2018/SriLanka spike CDS) showed higher similarities between α-CoV and β-CoV, ranging from 24.9 to 28.9%.

To sum up, the sequence differences between α-CoVs and β-CoVs of different subgenera were visualized with this comparative analysis. In general, we found highly conserved regions in the second part of the ORF1a CDS and the complete ORF1b CDS, which shared high identities among all analyzed α-CoV and β-CoV strains. The remaining parts of the ORF1a CDS and all the structural protein CDS showed higher diversity between α-CoVs and β-CoVs and also among each other.

### 3.2. Phylogeny

**Figure 3:**
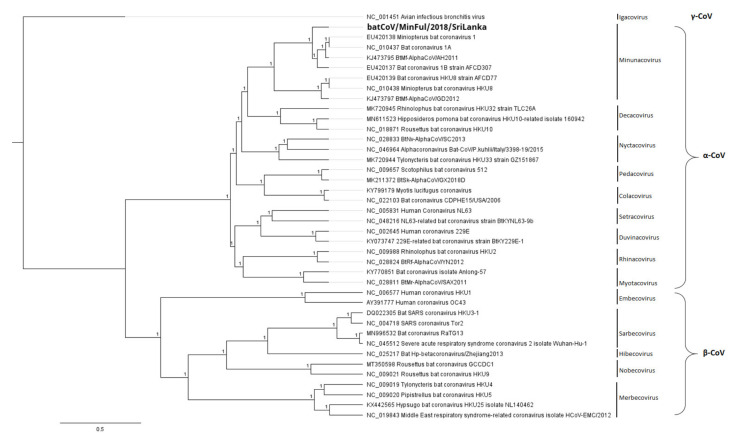
Phylogenetic tree based on a full genome nucleotide (nt) alignment of the novel strain batCoV/MinFul/2018/SriLanka (bold) with selected α-CoVs and β-CoVs and specification of the subgenera. The γ-CoV avian infectious bronchitis virus (NC_001451) was included as an outgroup for the calculation. The phylogenetic tree was calculated with Bayesian algorithm, and 500,000 generations were calculated with a subsampling frequency of 100 and a burn-in of 10%. Substitution model GTR was selected with a gamma-distributed rate variation.

A phylogenetic tree (Figure 3) was calculated for the full genome based on a nucleotide alignment with all selected reference sequences of the α-CoV and β-CoV subgenera. The calculation reveals the separation of these strains into distinct clades, α-CoVs and β-CoVs. In the α-CoV clade, strain batCoV/MinFul/2018/SriLanka clusters with other α-CoVs of the subgenus Minunacovirus. These are the strains that shared the highest overall pairwise identities with the novel batCoV/MinFul/2018/SriLanka strain on nt and aa level.

The remaining α-CoV strains form separate branches according to the assigned subgenera. For the β-CoV branch, the separation into the subgenera is clearly represented (compare Figure 3).

**Figure 4:**
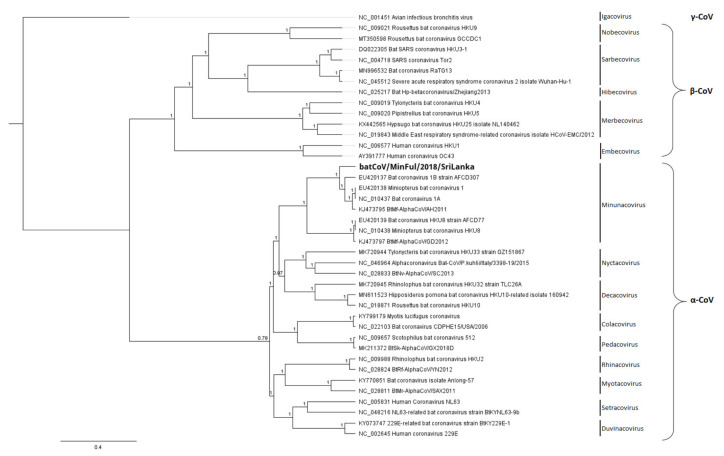
Phylogenetic tree based on an ORF1b nt alignment of the novel batCoV/MinFul/2018/SriLanka (bold) and selected CoV strains from different subgenera. The γ-CoV avian infectious bronchitis virus (NC_001451) was included as an outgroup for the calculation. The phylogenetic tree was calculated with the Bayesian algorithm, and 1 million generations were calculated with a subsampling frequency of 100 and a burn-in of 10%. Substitution model GTR was selected with a gamma-distributed rate variation.

**Figure 5:**
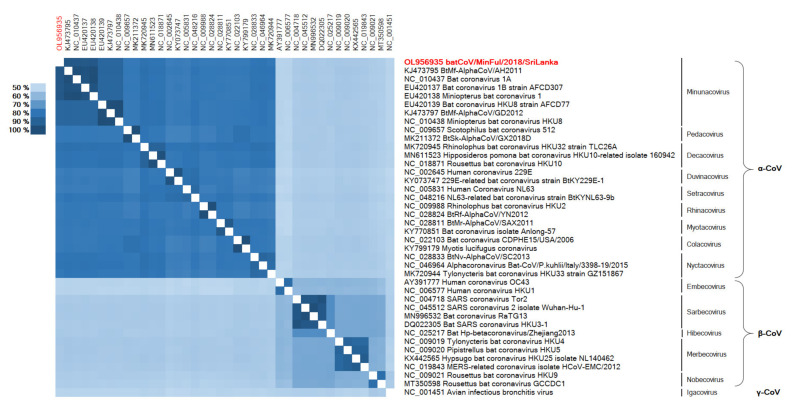
Heatmap based on an ORF1b aa alignment of the novel batCoV/MinFul/2018/SriLanka (red) and 39 selected CoV strains.

In addition to the phylogenetic reconstruction of the full genomes, the conserved ORF1b CDS was selected for phylogenetic analysis with further CoV strains. The phylogenetic tree (Figure 4) is based on a gap-free nucleotide alignment of the representative CoV strains of different subgenera, and the associated heatmap (Figure 5) displays the distances among these strains on an amino acid level.

In Figure 4 the phylogenetic reconstruction of batCoV/MinFul/2018/SriLanka ORF1b along with representative α-CoVs and β-CoVs is displayed. The γ-CoV strain NC_001451 (avian infectious bronchitis virus) was selected as the outgroup and used to root the phylogenetic tree. Within the β-CoVs clade, the separate subclades are visible, representing the subgenera Hibecovirus, Embecovirus, Sarbecovirus, Nobecovirus and Merbecovirus. These clusters are clearly visible in the associated heatmap as well (Figure 5). Within the α-CoV clade, the tree is partitioned into several subclades representing the subgenera, respectively. Inside the subclade of Minunacoviruses, the novel batCoV/MinFul/2018/SriLanka strain is clustered with α-CoV strains of the Miniopterus bat coronavirus 1 species. In this Minunacovirus subclade, the Miniopterus bat coronavirus HKU8-related strains build a separate branch. The distances between Miniopterus bat coronavirus 1 strains and HKU8 strains were confirmed with the associated heatmap as well (Figure 5).

### 3.3. Validation of NGS Results Using Real-Time RT-PCR

In order to validate the results of NGS data from the nine pool samples and test them back in the individual rectal swab samples, a specific real-time RT-PCR was designed. Specificity of the assay was tested with a number of other α-CoVs (HCoV-229E, -NL63) and β-CoVs (SARS-CoV, MERS-CoV, HCoVs-OC43). As no unspecific amplification was observed, a high specificity of the PCR assay to the novel batCoV/MinFul/2018/SriLanka can be assumed. All individual 65 rectal swabs from the analyzed NGS pools were tested with the newly designed real-time RT-PCR assay, and positive results were quantified by using a standardized synthetic RNA control (Table 4).

Overall, 36 out of the 65 rectal swabs (55%) tested positive for batCoV/MinFul/2018/SriLanka with viral loads of up to 2611 copies per reaction. The sensitivity of the newly designed real-time RT-PCR assay was 10 copies per reaction; therefore, all PCR-positive samples with concentrations of less than 15 copies per reaction were considered as uncertain positive results.

Of the 65 bats, the majority was female. To put this into context, of all 200 sampled bats that were sampled during the session in July 2018, a high number of 171 bats (85%) were females and only 29 captured bats were males.

In order to gain an overall prevalence of batCoV/MinFul/2018/SriLanka, we additionally tested all collected rectal swab and feces samples from all species and all time points with the specifically designed real-time RT-PCR (Table 5).

## 4. Discussion

In our study, we were able to obtain the full genome of an α-CoV strain named batCoV/MinFul/2018/SriLanka from *Miniopterus fuliginosus* bats from Sri Lanka. With our comparative analysis on the full genome level, we found overall high identities to the Miniopterus bat coronavirus 1, which belongs to the subgenus of Minunacoviruses within the α-CoVs. Therefore, we suggest that the detected batCoV/MinFul/2018/SriLanka belongs to the same subgenus. According to ICTV criteria, the demarcation threshold of a novel virus species within the coronavirus family is an aa identity of less than 90% in the conserved replicase domains [22,23]. With an identity of 95.5% to the reference strain BtMf-AlphaCoV/AH2011 (KJ473795), we therefore assume that the Sri Lankan strain belongs to the same virus species Miniopterus bat coronavirus 1.

The recombination analysis did not show signs of recombination between the novel batCoV/MinFul/2018/SriLanka and other strains of the Minunacovirus subgenus. The genetic comparison also showed that the novel batCoV/MinFul/2018/SriLanka has rather low identities to the human-pathogenic strains HCoVs 229E and NL63 compared to the other bat-related α-CoVs. For both HCoVs, different most common ancestors are assumed, namely a Hipposideros bat species for HCoV 229E and a Perimyotis bat species for HCoV NL63 [24].

In our study, we used the specifically designed rt-PCR to screen for the novel batCoV/MinFul/2018/SriLanka strain in all rectal swab and feces samples collected from Miniopterus, Hipposideros, Rhinolophus and Rousettus bats. Although all bats live in a sympatric colony in the Wavul Galge cave, the virus was detected only in *M. fuliginosus* bats. This supports the assumption that the batCoV/MinFul/2018/SriLanka strain has a high host specificity to Miniopterus bats; this also seems reasonable because of its high resemblance to Miniopterus-related viruses and lower similarity to other α-CoVs. This assumption is supported by the phylogenetic analysis where the branch of HCoVs is separated early from the remaining α-CoVs.

The high overall divergence between α-CoVs and β-CoVs was also demonstrated by means of the comparative and phylogenetic analysis. Again, this may be explained by the different natural hosts of α-CoVs and β-CoVs. Miniopterus bats and other species of the family Vespertilionidae are mainly known to carry α-CoVs, while β-CoVs are rather found in other Chiroptera families [25]. SARS-related β-CoVs are mainly found globally in Rhinolophus bats but were also detected in Hipposideros and Chaerephon bat species in Africa [25]. We reveal that the high diversity between the chiroptera-hosted CoVs may be explained by a host-related evolution of the viruses and is driven by geographically distinct bat species, which is in line with a prior study [26]. The phylogenetic trees likewise show an early separation of the α-CoV and β-CoV clades.

The main differences between the novel batCoV/MinFul/2018/SriLanka strain and other α-CoVs and β-CoVs are found in the structural proteins. Especially the amino acid sequences of spike, envelope and nucleocapsid showed the highest diversity among all strains. The spike protein is responsible for the virus attachment to the host cell and the fusion with the cellular membrane [7]. Since this process is very host specific, a high diversity of this protein can be assumed. The spike protein consists of two subunits, S1 and S2. The S1 subunit is the peripheral part of the protein and responsible for recognizing and binding to the host ACE2 receptor [24,27]. In detail, the affinity of the specific receptor binding domain (RBD) to this ACE2 receptor affects the efficiency of viral binding and entry to the host cell [28]. The RBD of human pathogenic CoVs is different to that of other chiroptera-hosted CoVs. In addition, the presence of a furin cleavage site in the spike protein is known to enhance the cell entrance of SARS-CoV-2 and other human pathogenic CoVs to the human host cell [29]. Comparing the spike protein sequences of the novel batCoV/MinFul/2018/SriLanka strain to different human pathogenic CoVs, we could not find the same RBD sequence. Furthermore, the furin cleavage motif was not present in the batCoV/MinFul/2018/SriLanka strain. With these sequence differences, a rather low human pathogenic potential may be concluded.

The S2 subunit is an integral membrane part of the spike protein and responsible for the fusion of the virus particle with the host membrane [27]. This process is rather independent of the respective host cell; therefore, the S2 subunit is more conserved among different CoV strains. In our analysis we confirmed this, showing that the S1 subunit of the spike protein is highly diverse, while the S2 subunit showed higher identities between all compared CoV strains.

In contrast to the structural proteins, the ORF1a/1b CDS are generally higher conserved parts of the genome. The complete ORF1a/1b polyprotein forms the largest part of the CoV genome and codes for different non-structural proteins for replication [25]. The highest resemblance among all compared CoV strains was observed in the ORF1b CDS, especially in the first part of the polyprotein coding for the RNA-dependent RNA polymerase [30]. In contrast, the ORF1a sequences seem to be less conserved and are different in particular between α-CoVs and β-CoVs. It has been discussed that the ORF1a part is not only responsible for replication but also important for the survival of the virus and its adaptation to the respective host [30]. Our analysis would support this assumption, since the compared CoV strains with a high diversity in ORF1a CDS originate from diverse hosts, while strains with the same host generally share higher identities in the OFR1a CDS as well.

In conclusion, a high host specificity of the novel batCoV/MinFul/2018/SriLanka to Miniopterus bat species can be assumed, while a risk of transmission from bats to humans is estimated to be rather low. In order to investigate possible transmission to other species, we used the newly designed real-time RT-PCR not only for the verification of the batCoV/MinFul/2018/SriLanka in the previously analyzed Miniopterus samples, but also for screening of all rectal swabs and feces samples that were collected during the three sampling sessions in March 2018, July 2018 and January 2019. With this extended screening we found an additional number of positive samples in all three sessions. Interestingly, these samples were only from Miniopterus bats, although we sampled bats of the genus Hipposideros, Rhinolophus and Rousettus in all sampling sessions. All these bat species live in a sympatric colony in the Wavul Galge cave (Sri Lanka) where the sampling took place. Although the roosting sites for each species are separated inside the cave, the bats have contact when exiting and entering the cave. In addition, urine droppings inside the cave would facilitate a transmission route via aerosols. Nevertheless, we did not find any indications for the presence of the virus in other bat species so far. This emphasizes that the virus is host specific for *M. fuliginosus* and transmission to other species is not very likely. There are no indications that an α-CoV like the batCoV/MinFul/2018/SriLanka strain has a high potential to be transmitted to humans and to cause a pandemic comparable to that of SARS-CoV-2. Nevertheless, follow-up studies with a higher number of all bat species in the Wavul Galge cave, blood collection to check for seroconversion and sampling at different time points over the year could verify the assumption of host specificity and check for seasonal shedding of the virus in *M. fuliginosus*.

## Figures and Tables

**Table 1:** Overview of α-Coronaviruses (-CoVs), β-CoVs and γ-CoV from the NCBI database that were selected for the genomic and phylogenetic analyses.

Genus	Subgenus	Accession No.	Description
α-CoV	Minunacovirus	KJ473795	BtMf-AlphaCoV/AH2011
		NC_010437	Bat coronavirus 1A
		EU420137	Bat coronavirus 1B strain AFCD307
		EU420138	Miniopterus bat coronavirus 1
		EU420139	Bat coronavirus HKU8 strain AFCD77
		KJ473797	BtMf-AlphaCoV/GD2012
		NC_010438	Miniopterus bat coronavirus HKU8
	Pedacovirus	NC_009657	Scotophilus bat coronavirus 512
		MK211372	BtSk-AlphaCoV/GX2018D
	Decacovirus	MK720945	Rhinolophus bat coronavirus HKU32 strain TLC26A
		MN611523	Hipposideros pomona bat coronavirus HKU10-related isolate 160942
		NC_018871	Rousettus bat coronavirus HKU10
	Duvinacovirus	NC_002645	Human coronavirus 229E
		KY073747	229E-related bat coronavirus strain BtKY229E-1
	Setracovirus	NC_005831	Human coronavirus NL63
		NC_048216	NL63-related bat coronavirus strain BtKYNL63-9b
	Rhinacovirus	NC_009988	Rhinolophus bat coronavirus HKU2
		NC_028824	BtRf-AlphaCoV/YN2012
	Myotacovirus	NC_028811	BtMr-AlphaCoV/SAX2011
		KY770851	Bat coronavirus isolate Anlong-57
	Colacovirus	NC_022103	Bat coronavirus CDPHE15/USA/2006
		KY799179	Myotis lucifugus coronavirus
	Nyctacovirus	NC_028833	BtNv-AlphaCoV/SC2013
		NC_046964	Alphacoronavirus bat-CoV/P.kuhlii/Italy/3398-19/2015
		MK720944	Tylonycteris bat coronavirus HKU33 strain GZ151867
β-CoV	Embecovirus	AY391777	Human coronavirus OC43
		NC_006577	Human coronavirus HKU1
	Sarbecovirus	NC_004718	SARS coronavirus Tor2
		NC_045512	SARS coronavirus 2 isolate Wuhan-Hu-1
		MN996532	Bat coronavirus RaTG13
		DQ022305	Bat SARS coronavirus HKU3-1
	Hibecovirus	NC_025217	Bat Hp-betacoronavirus/Zhejiang2013
	Merbecovirus	NC_009019	Tylonycteris bat coronavirus HKU4
		NC_009020	Pipistrellus bat coronavirus HKU5
		KX442565	Hypsugo bat coronavirus HKU25 isolate NL140462
		NC_019843	MERS-related coronavirus isolate HCoV-EMC/2012
	Nobecovirus	NC_009021	Rousettus bat coronavirus HKU9
		MT350598	Rousettus bat coronavirus GCCDC1
γ-CoV	Igacovirus	NC_001451	Avian infectious bronchitis virus

**Table 2:** Annotated coding sequences (CDS) of the novel batCoV/MinFul/2018/SriLanka strain compared to the reference genome KJ473795 (BtMf-AlphaCoV/AH2011).

	CDS	Start–End (Nucleotide Position)	No. of Nucleotides	No. of Amino Acids
batCoV/ MinFul/2018/ SriLanka	ORF1a	113–12,859	12,747	4249
	ORF1b	12,859–20,880	8022	2674
	Spike	20,882–25,009	4128	1376
	Envelope	25,662–25,886	225	75
	Membrane	25,893–26,651	759	253
	Nucleocapsid	26,672–27,841	1170	390
KJ473795	ORF1a	273–13,046	12,774	4258
	ORF1b	13,046–21,067	8022	2674
	Spike	21,069–25,196	4128	1376
	Envelope	25,849–26,073	225	75
	Membrane	26,080–26,841	762	251
	Nucleocapsid	26,862–28,031	1170	390

**Table 3:** Pairwise nucleotide (nt) and amino acid (aa) identities of the novel batCoV/MinFul/2018/SriLanka strain compared to selected α-CoVs and β-CoVs.

		Pairwise Nucleotide Identity (%)						Pairwise Amino Acid Identity (%)					
	Full Genome	ORF1a	ORF1b	S	E	M	N	ORF1a	ORF1b	S	E	M	N
**Alphacoronaviruses**													
Miniopterus bat coronavirus 1	84.9	83.0	89.2	83.1	92.4	88.1	88.5	86.1	94.9	87.1	91.9	88.7	89.5
BtMf-AlphaCoV/AH2011	85.1	83.2	89.7	83.2	92.9	86.5	89.2	86.6	95.5	87.7	91.9	87.5	98.7
BtMf-AlphaCoV/GD2012	66.8	65.5	77.7	62.5	70.7	73.9	58.0	63.3	87.4	59.1	67.6	74.5	57.2
Human coronavirus 229E	57.0	54.9	71.7	46.7	57.6	54.3	43.3	47.7	77.5	45.0	44.7	56.2	37.8
Human coronavirus NL63	59.4	55.7	73.3	52.8	61.5	56.0	49.3	47.7	77.6	43.5	51.3	60.6	45.5
**Betacoronaviruses**													
Bat SARS coronavirus HKU3-1	39.4	34.3	58.0	32.6	34.6	39.3	32.2	19.7	56.1	18.1	21.3	30.5	22.4
Rousettus bat coronavirus HKU9	39.1	33.2	57.9	34.0	41.5	43.6	30.6	19.7	55.8	18.3	15.6	31.6	18.5
Pipistrellus bat coronavirus HKU5	38.0	32.4	57.1	34.2	37.4	38.4	30.8	20.5	56.2	19.0	16.3	32.6	24.1
Human coronavirus HKU1	42.8	39.9	58.5	38.6	42.8	42.6	33.5	20.3	53.3	18.2	17.7	34.6	23.7
SARS-CoV-2 Wuhan-Hu-1	40.2	34.7	59.6	34.4	35.5	39.6	31.9	20.2	56.5	18.3	20.0	30.8	21.7
Human coronavirus OC43	40.3	39.0	57.3	37.7	40.3	41.1	32.0	20.3	53.2	18.1	17.9	32.5	21.0

ORF: open reading frame; S: spike CDS; E: envelope CDS; M: membrane CDS; N: nucleocapsid CDS.

**Table 4:** Overview of positive results after screening of the rectal swab with the newly designed real-time RT-PCR assay for the detection of the novel batCoV/MinFul/2018/SriLanka; copy numbers of 15 and below are shown in brackets.

Pool	Positive Samples/Total	Positive Sample	Sampling Date	Sex	Copies per Reaction (25 µL)
RS 2.2	6/8	RS 85	7 July 2018	M	(1)
		RS 91	7 July 2018	F	(14)
		RS 94	7 July 2018	M	(12)
		RS 95	7 July 2018	F	1313
		RS 96	7 July 2018	F	60
		RS 98	7 July 2018	F	468
RS 2.3	4/8	RS 106	7 July 2018	F	106
		RS 108	7 July 2018	F	371
		RS 109	7 July 2018	M	854
		RS 110	7 July 2018	F	1737
RS 2.4	4/7	RS 114	7 July 2018	F	70
		RS 117	7 July 2018	F	46
		RS 118	7 July 2018	F	(14)
		RS 119	7 July 2018	F	650
RS 2.5	3/7	RS 124	7 July 2018	F	105
		RS 126	7 July 2018	F	1391
		RS 133	7 July 2018	M	242
RS 2.6	3/6	RS 135	7 July 2018	F	339
		RS 137	7 July 2018	F	2124
		RS 138	7 July 2018	F	106
RS 2.7	2/7	RS 146	7 July 2018	F	44
		RS 147	7 July 2018	F	93
RS 2.8	5/9	RS 154	8 July 2018	F	2611
		RS 158	8 July 2018	F	71
		RS 159	8 July 2018	F	1196
		RS 162	8 July 2018	F	866
		RS 164	8 July 2018	F	33
RS 2.9	7/8	RS 168	8 July 2018	F	296
		RS 169	8 July 2018	F	76
		RS 170	8 July 2018	F	557
		RS 171	8 July 2018	M	196
		RS 172	8 July 2018	F	104
		RS 175	8 July 2018	F	464
		RS 176	8 July 2018	F	753
RS 2.10	2/5	RS 178	8 July 2018	F	600
		RS 187	8 July 2018	F	(5)

**Table 5:** Overview of the rectal swab and feces samples and pools per bat genus, collected at three different time points. Only samples from Miniopterus bats tested positive for the novel batCoV/MinFul/2018/SriLanka.

Genus	Miniopterus		Rousettus		Hipposideros		Rhinolophus	
	Rectal Swabs	Feces	Rectal Swabs	Feces	Rectal Swabs	Feces	Rectal Swabs	Feces
March 2018								
Pools	2	0	1	1	1	0	12	1
Positive samples	3/3	0/0	0/9	0/2	0/3	0/0	0/60	0/8
July 2018								
Pools	17	8	1	0	0	0	0	0
Positive samples	59/116	38/77	0/11	0/0	0/0	0/0	0/0	0/0
January 2019								
Pools	1	3	2	1	3	2	2	2
Positive samples	4/11	23/27	0/16	0/3	0/16	0/7	0/16	0/17
Total positive	66/130 (50%)	61/104 (58%)	0/36	0/5	0/19	0/7	0/76	0/25

## Data Availability

The data presented in this study are openly available in GenBank (https://www.ncbi.nlm.nih.gov/genbank/, accessed on 31 January 2022), Accession number OL956935.
